# Protein–Protein Interaction Network Analysis Reveals Several Diseases Highly Associated with Polycystic Ovarian Syndrome

**DOI:** 10.3390/ijms20122959

**Published:** 2019-06-18

**Authors:** Balqis Ramly, Nor Afiqah-Aleng, Zeti-Azura Mohamed-Hussein

**Affiliations:** 1 Centre for Bioinformatics Research, Institute of Systems Biology (INBIOSIS), Universiti Kebangsaan Malaysia, UKM Bangi 43600, Selangor, Malaysia; balqisramly@gmail.com (B.R.); afiqahaleng@gmail.com (N.A.-A.); 2 Centre for Frontier Sciences, Faculty of Science and Technology, Universiti Kebangsaan Malaysia, UKM Bangi 43600, Selangor, Malaysia

**Keywords:** polycystic ovarian syndrome, PCOS, PPI, MCODE, pathway, network, subnetwork

## Abstract

Based on clinical observations, women with polycystic ovarian syndrome (PCOS) are prone to developing several other diseases, such as metabolic and cardiovascular diseases. However, the molecular association between PCOS and these diseases remains poorly understood. Recent studies showed that the information from protein–protein interaction (PPI) network analysis are useful in understanding the disease association in detail. This study utilized this approach to deepen the knowledge on the association between PCOS and other diseases. A PPI network for PCOS was constructed using PCOS-related proteins (PCOSrp) obtained from PCOSBase. MCODE was used to identify highly connected regions in the PCOS network, known as subnetworks. These subnetworks represent protein families, where their molecular information is used to explain the association between PCOS and other diseases. Fisher’s exact test and comorbidity data were used to identify PCOS–disease subnetworks. Pathway enrichment analysis was performed on the PCOS–disease subnetworks to identify significant pathways that are highly involved in the PCOS–disease associations. Migraine, schizophrenia, depressive disorder, obesity, and hypertension, along with twelve other diseases, were identified to be highly associated with PCOS. The identification of significant pathways, such as ribosome biogenesis, antigen processing and presentation, and mitophagy, suggest their involvement in the association between PCOS and migraine, schizophrenia, and hypertension.

## 1. Introduction

Polycystic ovarian syndrome (PCOS) is a hormonal disorder that affects women in their reproductive years, and its cause remains unknown due to its heterogenic symptoms [1]. PCOS patients are often observed with concurrent health problems such as hypertension [2], type 2 diabetes mellitus [3], cardiovascular and cerebrovascular diseases [4], mental disorders [5], ovarian cancer and endometrial cancer [6], amongst others. However, the molecular mechanisms underlying the association between PCOS with these diseases remain unknown.

Novel insights into diseases and their relationships can be achieved through computational analysis on the integration of molecular data in comparing different diseases based on their shared interacting proteins. Diseases are classed as being comorbid based on the assumption that they share genetic components such as proteins and/or biological pathways [7]. Shared proteins can be identified from protein–protein interaction (PPI) network analysis. PPI is often used to elucidate the molecular basis of diseases and providing detailed knowledge on the proteins and their interaction that can be used to suggest and improve diagnosis, prevention, and treatment of the diseases [8,9]. PPI network analysis provides information on shared genes and proteins in diseases to depict the interactions [10]. Several studies have demonstrated the use of the PPI network approach in understanding human diseases [9,11,12,13].

Here, a similar approach was applied to investigate the association between PCOS and other diseases. We used the MCODE algorithm to identify highly connected regions in the PCOS PPI network that represent molecular complexes. These complexes were used to discover shared proteins and shared pathways between PCOS and its associated diseases.

## 2. Results

### 2.1. Protein–Protein Interaction Network of PCOS

In total, 8185 PCOS-related proteins (PCOSrps) were used to build the PCOS PPI network. Overall. 20,277 interactions were established between 5213 PCOSrps. PPI information was obtained from the Human Integrated Protein–Protein Interaction Reference (HIPPIE) database. The remaining 2972 PCOSrps were excluded from the network due to the unavailability of their interaction partners and significant interactions calculated by HIPPIE. This dataset was used in the two-tier analysis, i.e., to search for diseases associated with PCOS using subnetworks (described as PCOS–disease subnetworks) and to identify all significant pathways in describing PCOS–disease associations (refer to pathway enrichment analysis).

### 2.2. PCOS–Disease Subnetwork

**Figure 1:**
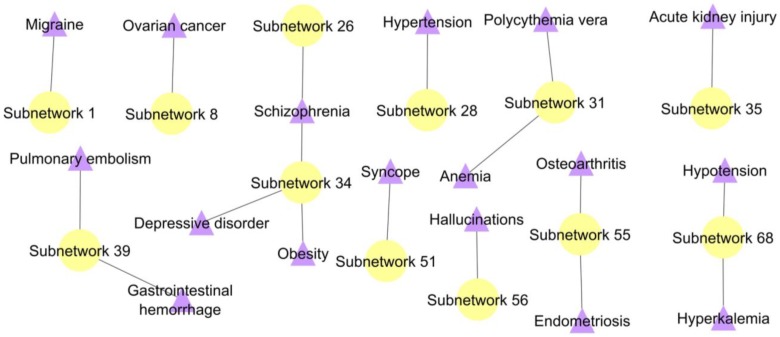
A disease subnetwork highlights significant diseases associated with polycystic ovarian syndrome (PCOS). Shared PCOSrp-enriched subnetworks were evaluated using Fisher’s exact test for their significance. Yellow node represents shared PCOSrp-enriched subnetwork and purple node refers to significant disease.

The MCODE algorithm with default parameters (node score cut-off = 0.2, degree cut-off = 2, k-core = 2, maximum depth set at 100) identified 77 PPI subnetworks in the PCOS network (Table A1). Overall, 17 significant diseases that co-occur with PCOS were identified from the 12 PPI subnetworks based on the *p*-value < 0.01 calculated from the Fisher’s exact test (Figure 1). The association between PCOS and other diseases were discovered from the identification of proteins that occur in both diseases (we named them as shared proteins); i.e., the participation/presence of PCOSrps in other diseases. Some PCOSrps have been identified in other diseases such as migraine, ovarian cancer, schizophrenia, hypertension, and depressive disorder, along with twelve other diseases.

Subnetwork 1 highlighted the association of PCOS with migraine, in which three PCOSrps were found in PCOS and migraine. These were 40S ribosomal proteins, namely RPS7, RPS10, and RPS26, which are involved in ribosome biogenesis.

In PCOS–disease subnetwork 8, five PCOSrps were found for ovarian cancer, suggesting the association between PCOS and ovarian cancers. Those proteins were BRCA1 (breast cancer type 1 susceptibility protein), CDKN1B (cyclin-dependent kinase inhibitor 1B), PPP1CC (serine/threonine-protein phosphatase PP1-gamma catalytic subunit), URI1 (unconventional prefoldin RPB5 interactor 1), and SKP2 (s-phase kinase-associated protein 2).

Subnetworks 26 and 34 suggested the association between PCOS and schizophrenia with the identification/existence of four PCOSrps (HLA-A (HLA class I histocompatibility antigen, A-2 alpha chain), HLA-C (HLA class I histocompatibility antigen, Cw-12 alpha chain), HLA-E (HLA class I histocompatibility antigen, alpha chain E), and LILRB1 (leukocyte immunoglobulin-like receptor subfamily B member 1)) in subnetwork 26 and 27 PCOSrps in subnetwork 34. Further, these proteins were found in schizophrenic patients, as listed in DisGeNET [14]. The PPI subnetwork analysis also suggested the association between PCOS and depressive disorder, as well as obesity, with the identification of 13 PCOSrps in depressive disorder and eight PCOSrps found in obesity. One PCOSrp (RAC-alpha serine/threonine-protein kinase, AKT1) was found to be involved in schizophrenia, depressive disorder, and obesity.

The association between PCOS and hypertension was discovered from subnetwork 28 with the identification of five PCOSrps; i.e., BBS1 (Bardet–Biedl syndrome 1 protein), BBS2 (Bardet–Biedl syndrome 2 protein), JUN (transcription factor AP-1), MYH9 (myosin-9), and SMAD4 (mothers against decapentaplegic homolog 4), and all these are linked to hypertension. Significant subnetworks with shared PCOSrps are presented in Table 1.

### 2.3. Pathway Enrichment Analysis

Pathway enrichment analysis was performed to find pathways that are statistically involved in PCOS and its associated diseases. The significant pathway is referred to the same pathway involved in both PCOS and its associated diseases. Information on the PCOS–disease association and their interacting shared proteins provides the opportunity to recognize potentially interesting gene and protein candidates that can be used to investigate the genetic basis of PCOS.

**Figure 2:**
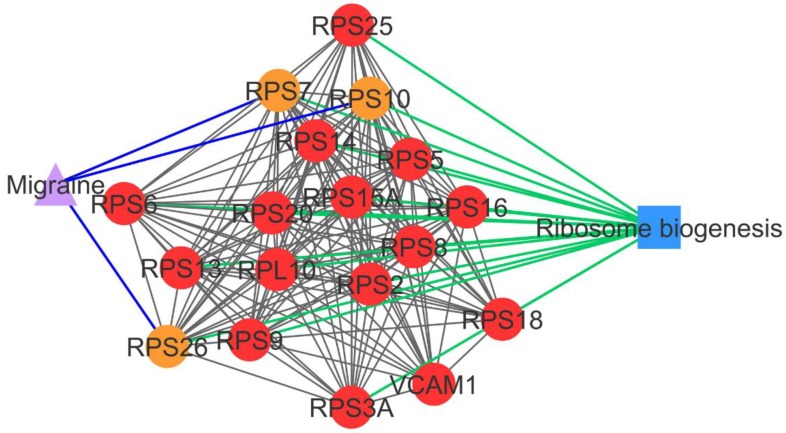
PCOS–disease subnetwork 1. This subnetwork highlights shared proteins and one shared pathway (ribosome biogenesis) between PCOS and migraine. Different coloring of nodes and lines represents PCOS-related proteins (red), shared proteins (orange), PCOS–disease interactions (blue), and protein–pathway interactions (green). Circle nodes refer to PCOS-related proteins, triangle nodes refer to disease, and square nodes refer to pathway.

**Figure 3:**
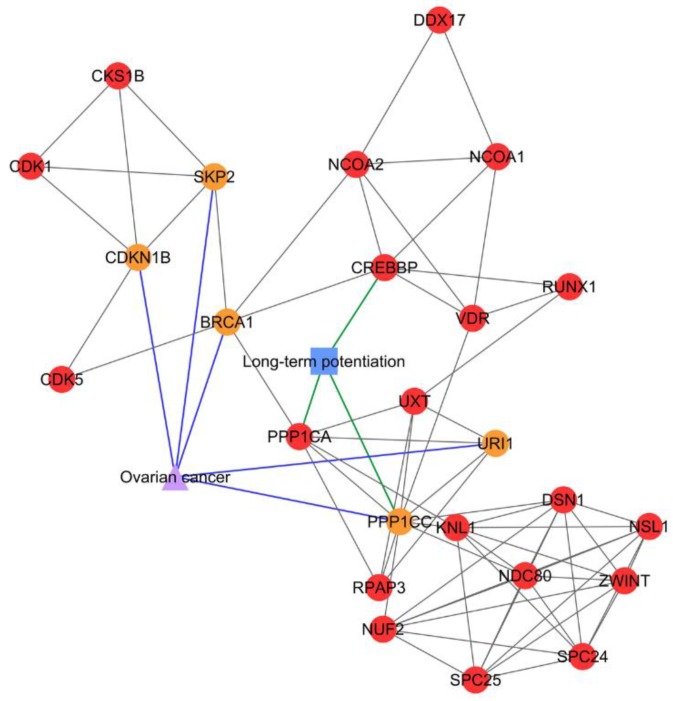
PCOS–disease subnetwork 8. This subnetwork showed shared proteins and shared pathway (long-term potentiation) between PCOS and ovarian cancer. Different coloring of nodes and lines represents PCOS-related proteins (red), shared proteins (orange), PCOS–disease interactions (blue), and protein–pathway interactions (green). Shape of nodes denotes PCOS-related proteins (circle), disease (triangle), and pathway (square).

Subnetwork 1 was enriched with ribosomal proteins that are involved in ribosome biogenesis or protein translation (Figure 2). In subnetwork 8, long-term potentiation was identified as a significant pathway, where PPP1CC and PPP1CA directly interacted with three PCOS-ovarian cancer-shared PCOSrps—i.e., BRCA1, PPP1CC, and URI1—and indirectly interacted with two shared PCOSrps (CDKN1B and SKP2; Figure 3).

**Figure 4:**
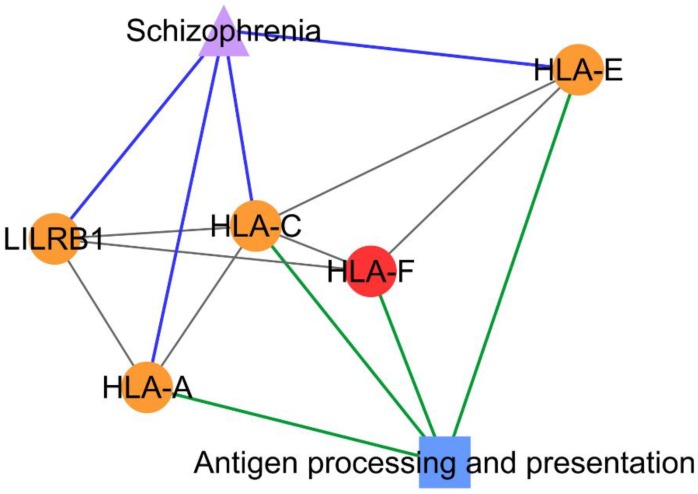
PCOS–disease subnetwork 26. Shared proteins and shared pathway (antigen processing and presentation) between PCOS and schizophrenia were identified from this subnetwork. Different coloring of nodes and lines represents PCOS-related proteins (red), shared proteins (orange), PCOS–disease interactions (blue), and protein–pathway interactions (green). Shape of nodes denotes PCOS-related proteins (circle), disease (triangle), and pathway (square).

From the pathway enrichment analysis, subnetwork 26 was enriched with proteins involved in an antigen processing and presentation pathway that consisted of three PCOSrps (HLA-A, HLA-C, and HLA-E) shared between PCOS and schizophrenia (Figure 4).

**Figure 5:**
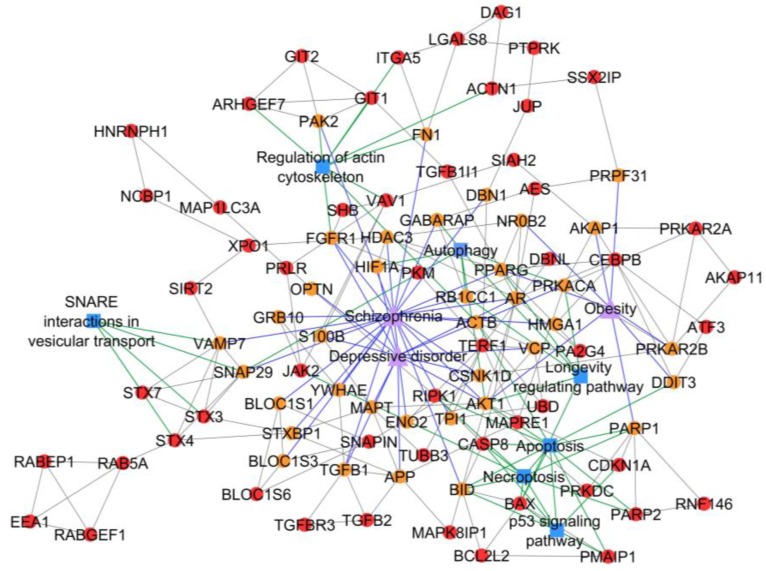
PCOS–disease subnetwork 34. This subnetwork highlighted shared proteins and seven shared pathways between PCOS and schizophrenia, depressive disorder, and obesity. Different coloring of nodes and lines represents PCOS-related proteins (red), shared proteins (orange), PCOS–disease interactions (blue), and protein–pathway interactions (green). Shape of nodes denotes PCOS-related proteins (circle), diseases (triangle), and pathways (square).

**Figure 6:**
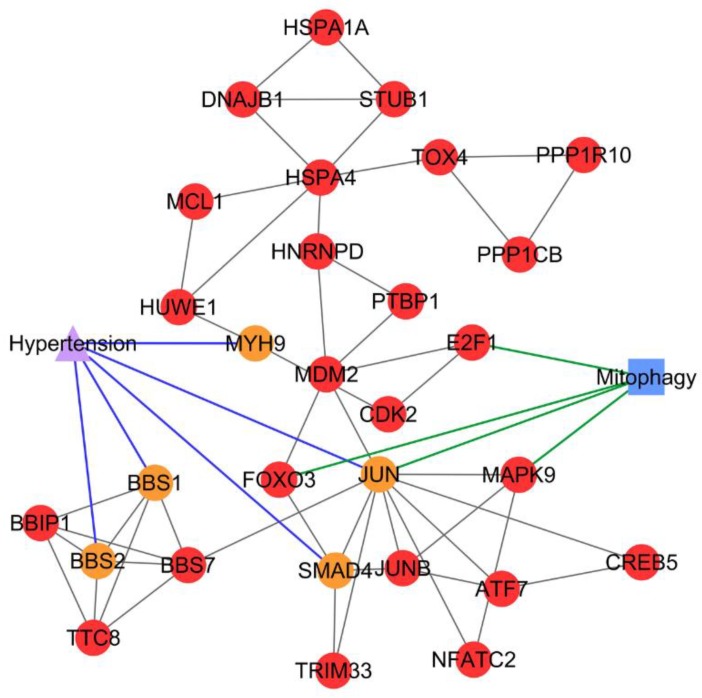
PCOS–disease subnetwork 28. Shared proteins and shared pathway (mitophagy) between PCOS and hypertension were identified from this subnetwork. Different coloring of nodes and lines represents PCOS-related proteins (red), shared proteins (orange), PCOS–disease interactions (blue), and protein–pathway interactions (green). Circle nodes refer to PCOS-related proteins, triangle node refers to disease, and square node refers to pathway.

Meanwhile, autophagy, apoptosis, and necroptosis were identified as significant pathways in subnetwork 34, which refers to the association of PCOS with schizophrenia, depressive disorder, and obesity (Figure 5). The PCOS-hypertension association was identified from subnetwork 28, where mitophagy was a significant shared pathway between PCOS and hypertension (Figure 6). Shared pathways for all identified PCOS–disease subnetworks are listed in Table 2. On the other hand, four PCOS–disease subnetworks did not have any significant pathways. Possible reasons for this include: (a) the PCOSrps in those subnetworks are involved in different pathways, (b) there is no pathway information related to PCOSrps in the subnetworks, and (c) outdated or incomplete gene and protein annotations.

## 3. Discussion

In this study, we successfully identified 17 diseases from 12 PCOS–disease subnetworks using the MCODE algorithm. The diseases were significantly related to PCOS based on the Fisher’s exact test calculated *p*-value of <0.001, and they were also found from clinical observations on PCOS women [15]. The association between PCOS and other diseases was identified using shared PCOSrps (the existence of PCOS-related proteins in other diseases) in the PCOS–disease subnetworks. Pathway analysis on the subnetworks has identified shared pathways between diseases. This information describes the association between PCOS and other diseases. Interestingly, some diseases that were known to be comorbid with PCOS were not identified; for example, type 2 diabetes mellitus and cerebrovascular diseases, even though both were reported by the patients [3,4]. This is due to the insignificant values in the subnetworks formed by the interactions between shared PCOSrps in those diseases.

PCOSrps RPS7, RPS10, and RPS26 were found in PCOS–disease subnetwork 1. These proteins were categorized as shared proteins and might play a role in PCOS–migraine association. RPS7, RPS10, and RPS26 are ribosomal proteins that play a role in ribosome assembly and protein translation [16]. This pathway was categorized as a significant pathway in subnetwork 1, in consensus with the results of Polzikov et al., where they found that ribosomal proteins were significantly upregulated in the cumulus cells of women with PCOS [17]. Genes involved in the ribosome pathway were highly expressed in the blood of migraine patients [18]. The dysregulation of ribosome biogenesis associates with cell proliferation, where the abnormal cell proliferation is commonly found in the cumulus and granulosa cells of women with PCOS [19], and the induction of stem cell proliferation was detected in migraine patients [20].

BRCA1, CDKN1B, PPP1CC, SKP2, and URI1 were clustered in subnetwork 8 and categorized as shared proteins in PCOS and ovarian cancer, hence suggesting an association between the two. This subnetwork was enriched with the long-term potentiation pathway that is involved in synaptic efficacy and plays a role in learning and memory [21]. Long-term potentiation was found to be correlated with estrogen [22]. Elevated levels of estrogen are commonly detected in women with PCOS [23] and ovarian cancer [24], thus enhancing long-term potentiation activity.

The association between PCOS and schizophrenia was identified in subnetwork 26 and subnetwork 34. The antigen processing and presentation pathway was significant in subnetwork 26, as the majority of the proteins in this subnetwork consist of HLA proteins, also known as major histocompatibility complex (MHC) proteins, which are main players in this pathway. This pathway was also involved in autoimmune diseases and immune reaction [21]. This result is supported by those of Li et al. from their epigenome-wide association study (EWAS), where DNA methylation profiling suggested the existence of an autoimmune basis in the pathogenesis of PCOS [25]. The antigen processing and presentation pathway was detected in schizophrenia patients based on the finding in the HLA region and from genome-wide association studies (GWAS) [26,27]. There is also increasing evidence for the involvement of MHC in the pathogenesis of schizophrenia, such as neurogenesis, neuronal differentiation and migration, and synaptic plasticity [28]. All these associations suggest the possibility of antigen processing and presentation as one of the shared pathways between PCOS and schizophrenia.

PCOS–disease subnetwork 34 not only showed the association between PCOS and schizophrenia, but also displayed the association between PCOS, depressive disorder, and obesity. This finding suggests that schizophrenia, depressive disorder, and obesity can simultaneously occur in women with PCOS. This association exists if there is a perturbation in autophagy, which is one of the enriched pathways in subnetwork 34. Autophagy is a degradation process of cytoplasmic components mediated by lysosomes, involving four shared PCOSrps (AKT1, RB1CC1 (RB1-inducible coiled-coil protein 1), GABARAP (gamma-aminobutyric acid receptor-associated protein), and SNAP29 (synaptosomal-associated protein 29)) with schizophrenia, three shared PCOSrps (AKT1, HIF1A (hypoxia-inducible factor 1-alpha), and PRKACA (cAMP-dependent protein kinase catalytic subunit alpha)) with depressive disorder, and one shared PCOrp (AKT1) with obesity. Any interaction between PCOSrps involved in autophagy with AKT1, be it direct or indirect, is strongly associated with the development of these comorbidities. The autophagy-related genes were significantly downregulated in PCOS endometrial tissue [29] as found by Li et al. in their study on the dysregulation of autophagy in PCOS, where it was abnormally enhanced in both human and rat PCOS ovarian tissue [30]. Meanwhile, elevated insulin levels and/or insulin resistance are commonly seen in women with PCOS, and this is one of the most important mechanisms in PCOS pathogenesis [31,32]. It was found to be able to impair ovarian autophagy and function in mice [33]. Recently, insulin-sensitizers such as inositol have been used to improve the insulin resistance in PCOS by regulating autophagy [34]. On the other hand, transcriptional profiling in schizophrenia patients showed the connection of autophagy dysregulation during a malfunction of autophagy-related genes in Brodmann Area 22 [35]. The brain tissues of patients with depressive disorder demonstrated the increase of apoptotic stress and apoptotic-related factors [36,37], suggesting the role of autophagy impairment in depressive disorder. Energy imbalance and neurohormonal dysregulation in obese patients were found to be tightly regulated by autophagy, hence suggesting its vital role in the pathophysiology of obesity [38]. Autophagy impairment is connected to the PI3K-Akt-mTOR signaling pathway and it is also associated with (i) PCOS, as it regulates the androgens, insulin, and insulin-like growth factors [39]; (ii) mental illnesses, as it links to the protein synthesis in synapses [40]; and (iii) obesity, as it is activated by excessive nutrition [30]. Pathway enrichment analysis on PCOS–disease subnetwork 34 identified apoptosis and necroptosis as shared pathways between PCOS and schizophrenia, depressive disorder, and obesity. Our finding corroborate with the findings from others [41], suggesting crosstalk between these pathways in facilitating cell destruction.

Results of the pathway enrichment analysis of PCOS–disease subnetwork 28 suggested the comorbidity between hypertension and PCOS, as both diseases shared the mitophagy pathway. Four PCOSrps were identified to be involved in mitophagy, i.e., E2F1 (transcription factor E2F1), FOXO3 (forkhead box protein O3), JUN, and MAPK9 (mitogen-activated protein kinase 9). Mitophagy is a process of damaged mitochondria removal via autophagy [42]. Mitochondria are essential organelles in the oocyte, which play a critical role in oocyte maturation, fertilization, and embryo development [43,44]. If mitochondria are disrupted by mitophagy impairment, PCOS oocyte structures will be deformed [43]. This leads to the production of poor-quality oocytes in women with PCOS [44], and hence it will lower the fertilization rate of women with PCOS undergoing in vitro fertilization (IVF), even though the oocyte retrieval is higher [45]. Mitophagy impairment contributes to mitochondrial abnormalities and dysfunction in cardiovascular homeostasis. These conditions are found in cardiomyocytes of hypertensive rats [46]. Eisenberg et al. demonstrated the connection between mitophagy and hypertension, where dietary spermidine can lower the blood pressure and enhance cardiac mitophagy in hypertensive rats by eliciting cardioprotective effects [47]. JUN (a PCOSrp) plays a role in mitophagy, and it was also identified as a shared protein between PCOS and hypertension. These findings suggest the possibility of PCOS association with high blood pressure.

Findings from this study demonstrate that the integration of human PPI networks with protein–disease information and pathway enrichment analysis of the PCOS–disease network can be used as a proof-of-principle, where only several subnetworks were discussed as examples to describe the association of PCOS with 17 diseases. Important shared proteins and pathways between PCOS and specific associated diseases were identified, and further experimental studies need to be carried out to validate the obtained information that will provide new insights into molecular mechanisms of PCOS. A better understanding of the pathophysiology of PCOS will be essential for the management of PCOS and its complications. Nonetheless, these novel relationships could offer new insights into disease etiology and classification, as well assisting several aspects such as biomarker development, drug target discovery, and diagnosis improvement.

## 4. Materials and Methods

### 4.1. Compilation of PCOS-Related Proteins and Their Associated Diseases

PCOS-related proteins (PCOSrps) and their associated diseases were retrieved from PCOSBase (PCOSBase v1.0; www.pcosbase.org) [48], a manually curated medically oriented database. PCOSBase compiles PCOSrp from nine protein–disease association databases (i.e., DisGeNET [14], DISEASES [49], Disease and Gene Annotation (DGA) [50], GWAS Catalog [51], GWASdb [52], MalaCards [53], Online Mendelian Inheritance in Man (OMIM) [54], PhenomicDB [55], and The Human Gene Mutation Database (HGMD) [56] and 31 gene and protein expression studies. Meanwhile, all diseases associated with PCOSrps that were listed in PCOSBase were retrieved from DisGeNET (www.disgenet.org) [14].

### 4.2. Construction of PCOS PPI Network

A PCOS PPI network was constructed using a PCOSrp dataset obtained from PCOSBase, combined with the information on PCOSrps that were obtained from Human Integrated Protein–protein Interaction Reference (HIPPIE) (http://cbdm-01.zdv.uni-mainz.de/~mschaefer/hippie/) [57]. HIPPIE scores of ≥0.73 were chosen to ensure the reliability of interactions between proteins. Cytoscape v3.6.0 was used to construct and visualize the network [58].

### 4.3. Construction of PCOS–Disease Subnetworks

The MCODE algorithm [59], one of the Cytoscape v3.6.0 plugins, was used to find highly interconnected regions or those of high density in the PCOS network. Density of a subnetwork is a ratio of the number of subnetwork edges (|E|) and the maximum possible number of cluster edges (|E|_max_ = |V|(|V| − 1)/2, where V is the number of nodes in the subnetwork). Density and number of nodes are used to calculate the score of each subnetwork (score = density × number of nodes) [59]. PPI subnetworks with more than two interactions were filtered (core > 2).

Each protein in a PPI subnetwork was annotated with disease-associated information compiled from DisGeNET [14]. Fisher’s exact test was performed to identify diseases that were significantly (*p*-value < 0.01) associated with PCOS in every PPI subnetwork. This statistical significance test used the analysis of 2 x 2 contingency tables [60,61]. The values of a, b, c, and d were determined for each disease in the subnetwork, as demonstrated in Table 3.

Comorbidity data obtained from [15] supported the association between PCOS and its associated diseases as predicted in this study. PPI subnetworks that contained significantly associated diseases comorbid with PCOS women were categorized as PCOS–disease subnetworks and chosen for the pathway enrichment analysis in order to identify shared pathways between PCOS and its associated diseases. PCOSrps and PCOS-associated diseases were represented in different shapes of nodes in the PCOS–disease subnetwork.

### 4.4. Pathway Enrichment Analysis

**Figure 7:**
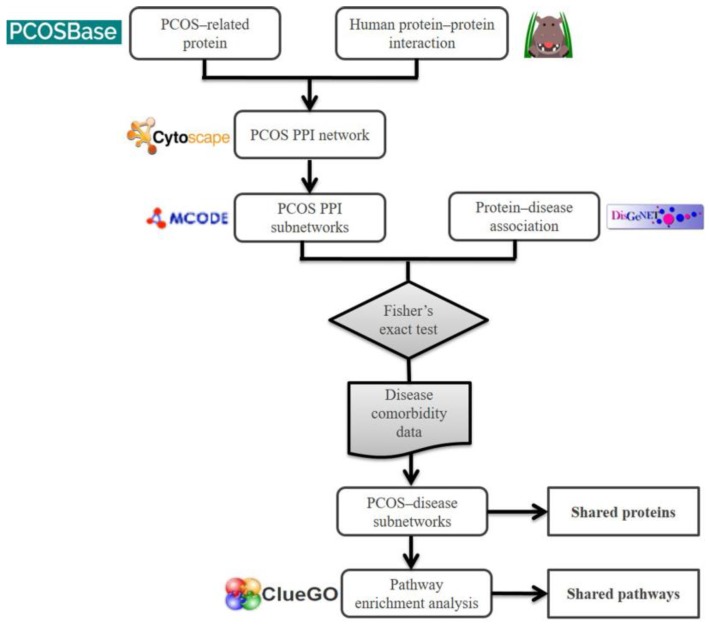
Framework of the computational method used to elucidate the association of PCOS with other diseases.

The biological function of the subnetworks were determined from the pathway enrichment analysis using ClueGO [62] against the Kyoto Encyclopedia of Genes and Genomes (KEGG) database [21]. ClueGO was used to identify shared pathways in PCOS and its associated diseases from the PCOS–disease subnetworks. Shared pathways were identified using a hypergeometric test followed by the application of Bonferroni stepdown to calculate the false discovery rate (FDR). Overall method of this study is illustrated in Figure 7.

## Figures and Tables

**Table 1:** Significant PCOS–disease subnetworks. Significant subnetworks with shared PCOSrps, suggesting diseases association with PCOS.

PCOS–Disease Subnetwork	Density Score	Number of Nodes	Number of Interactions	PCOS-Associated Disease	Shared PCOSrps
1	17.882	18	152	Migraine	RPS7, RPS10, RPS26
8	5.5	25	66	Ovarian cancer	BRCA1, CDKN1B, PPP1CC, SKP2, URI1
26	3.5	5	7	Schizophrenia	HLA-A, HLA-C, HLA-E, LILRB1
28	3.357	29	47	Hypertension	BBS1, BBS2, JUN, MYH9, SMAD4
31	3.333	4	5	Anemia	HBA1, HBBHBA1, HBB
				Polycythemia vera	
34	3.067	91	138	Schizophrenia	ACTB, AKT1, AR, BID, BLOC1S3, CSNK1D, DBN1, ENO2, FGFR1, FN1, GABARAP, GRB10, HDAC3, HMGA1, PAK2, PPARG, RB1CC1, S100B, SNAP29, TGFB1, TPI1, VAMP7, YWHAE
				Depressive disorder	AKT1, APP, AR, ATF3, CSNK1D, FGFR1, HIF1A, MAPT, OPTN, PRKACA, S100B, TGFB1, VCP
				Obesity	AKAP1, AKT1, DDIT3, NR0B2, PARP1, PPARG, PRKAR2B, PRPF31
35	3.043	24	35	Acute kidney injury	EGFR, GSTM2, TP53
39	3	9	12	Gastrointestinal hemorrhage	FGA, FGB, FGG
				Pulmonary embolism	FGA, PLAT
51	3	3	3	Syncope	CACNA1C, RYR2
55	3	3	3	Endometriosis	AKR1C1, AKR1C2, AKR1C3
				Osteoarthritis	AKR1C1, AKR1C2
56	3	3	3	Hallucinations	BCKDHA, BCKDHB
68	3	3	3	Hyperkalemia	SCNN1A, SCNN1G
				Hypotension	SCNN1A, SCNN1G

**Table 2:** Shared PCOS–disease pathways. These pathways were shared between PCOS and respective diseases identified from pathway enrichment analysis.

PCOS–Disease Subnetwork	PCOS-Associated Disease	Shared Pathway
1	Migraine	Ribosome
8	Ovarian cancer	Long-term potentiation
26	Schizophrenia	Antigen processing and presentation
28	Hypertension	Mitophagy
31	Anemia and polycythemia vera	No enriched pathway
34	Schizophrenia, depressive disorder, and obesity	Longevity regulation pathway, necroptosis, apoptosis, regulation of actin cytoskeleton, autophagy, p53 signaling pathway, and SNARE interactions in vesicular transport
35	Acute kidney injury	Adherens junction, glutathione metabolism
39	Pulmonary embolism and gastrointestinal hemorrhage	Compliment and coagulation cascades
51	Syncope	No enriched pathway
55	Endometriosis and osteoarthritis	Steroid hormone biosynthesis
56	Hallucinations	No enriched pathway
68	Hyperkalemia and hypotension	No enriched pathway

**Table 3:** 2 × 2 contingency table. The values of a, b, c and d in the table were used in Fisher’s exact test.

	Shared PCOSrps	Non-shared PCOSrps	
In subnetwork	a	b	a + b
Outside subnetwork	c	d	c + d
	a + c	b + d	*n* ^1^

^1^ n is the total number of proteins in the PCOS protein–protein interaction (PPI) network.

## Abbreviations

DGA	Disease and gene annotation
EWAS	Epigenome-wide association study
FDR	False discovery rate
GWAS	Genome-wide association study
HGMD	Human Gene Mutation Database
HIPPIE	Human Integrated Protein–protein Interaction Reference
IVF	In vitro fertilization
KEGG	Kyoto Encyclopedia of Genes and Genomes
MHC	Major histocompatibility complex
OMIM	Online Mendelian Inheritance in Man
PCOS	Polycystic ovarian syndrome
PCOSrp	PCOS-related protein
PPI	Protein–protein interaction

## Appendix A

**Table A1:** Subnetworks generated from MCODE.

Cluster	Score (Density × #Nodes)	#Nodes	Edges	PCOSrps
1	17.882	18	152	RPL10, RPS10, RPS13, RPS14, RPS15A, RPS16, RPS18, RPS2, RPS20, RPS25, RPS26, RPS3A, RPS5, RPS6, RPS7, RPS8, RPS9, VCAM1
2	15.4	41	308	ADRM1, CCDC74B, CDK19, CDK8, ESR1, MED12, MED14, MED15, MED17, MED19, MED20, MED21, MED30, MED4, MED6, MED7, MED9, PAAF1, POMP, PSMA1, PSMA2, PSMA3, PSMA4, PSMB1, PSMB3, PSMB4, PSMB5, PSMB8, PSMB9, PSMC1, PSMC2, PSMC3, PSMC4, PSMC6, PSMD10, PSMD11, PSMD12, PSMD2, PSMD3, PSMD7, UCHL5
3	8	9	32	C1D, DIS3, EXOSC2, EXOSC3, EXOSC4, EXOSC6, EXOSC7, EXOSC8, EXOSC9
4	7.607	57	213	ACTL6A, ARID1A, ARID1B, CCNA1, CCT2, CCT3, CCT6A, CCT7, CCT8, CDK2AP1, CDT1, CHD4, DPF3, EPC1, FZR1, GATAD2A, IGBP1, ING3, INO80B, INO80C, INO80D, INO80E, MBD3, MCM2, MCM3, MCM4, MCM5, MCM6, MCM7, MORF4L1, MORF4L2, MTA1, NFRKB, PLK1, POLR2A, POLR2B, POLR2D, POLR2E, POLR2G, POLR2J, PPP2CB, PPP4C, RBBP4, RUVBL1, RUVBL2, SLMAP, SMARCA2, SMARCC1, SMARCC2, SMARCD1, SSRP1, STK24, STRN3, STRN4, TCP1, YEATS4, YY1
5	6.333	7	19	BRD4, CHTF18, DSCC1, RFC2, RFC3, RFC4, RFC5
6	6	7	18	SUPT3H, SUPT7L, TADA1, TADA2B, TADA3, TAF12, USP22
7	6	7	18	LSM2, LSM3, LSM4, LSM5, LSM6, LSM7, USP15
8	5.5	25	66	BRCA1, CDK1, CDK5, CDKN1B, CKS1B, CREBBP, DDX17, DSN1, KNL1, NCOA1, NCOA2, NDC80, NSL1, NUF2, PPP1CA, PPP1CC, RPAP3, RUNX1, SKP2, SPC24, SPC25, URI1, UXT, VDR, ZWINT
9	5	5	10	RPL13, RPL21, RPL36, RPL7A, RPLP0
10	5	5	10	NUP37, NUP43, NUP85, SEC13, SEH1L
11	4.857	8	17	CCDC93, COMMD1, COMMD10, COMMD2, COMMD4, COMMD6, ELOC, SOCS1
12	4.8	6	12	ABI1, ABI2, CYFIP1, CYFIP2, WASF2, ZNF511
13	4.571	8	16	MRPL1, MRPL12, MRPL42, MRPL44, MRPL50, MRPL51, MRPL55, MRPL58
14	4.5	5	9	CTTNBP2NL, FGFR1OP2, PDCD10, PPP2R1A, STRIP1
15	4.259	55	115	ARMC8, BARD1, BCL3, BCLAF1, BRCA2, BRCC3, CARM1, CDCA5, CLK2, CTNNB1, DDX5, FOS, GPS2, ILF2, LUC7L2, MAEA, MET, MRE11, NBN, NCOA3, NDRG1, NOP2, NOP56, NR3C1, NXF1, PDS5A, PDS5B, PGR, PPARGC1A, PRG2, RAD21, RAD50, RANBP2, RANBP9, RELA, RNPS1, RPL14, RPL15, RPL18, RPL18A, RPL23A, RPL24, RPL26, RPL37A, SMC1A, SMC3, SRPK2, SRRM1, SRRM2, SUMO3, TBL1X, TBL1XR1, TRA2A, TRIM28, WAPL
16	4.027	76	151	ACTR1B, ACTR3, ARPC2, ARPC3, ARPC4, ATF4, BHLHE40, BORCS6, CBLB, CEBPG, CFLAR, CSPP1, DCTN1, DCTN2, DCTN5, DCTN6, DNMT1, DNMT3B, EAF1, ERBB3, ETS1, EWSR1, EZR, FADD, FAS, FOSL1, GADD45A, GADD45G, GPR137B, HAUS1, HAUS2, HAUS4, HAUS5, HAUS6, HAUS7, HDAC1, HDAC2, IDI2, INSR, KDM1A, LAMTOR1, LAMTOR2, LAMTOR3, LAMTOR4, LMO4, LUC7L, MAF, MAP2K1, MAP2K2, MAPK3, NCAPD2, NCAPG, NCAPH, NCOR2, NPM1, PIK3R1, PRMT1, PTMA, PTPN11, RAF1, RRAGC, SMC2, SMC4, SOCS3, SSBP2, SSBP3, SSBP4, SUZ12, SYK, TAF1D, TDG, TOP2A, TRADD, UBE2I, ZEB2, ZHX1
17	4	4	6	GSE1, HMG20A, HMG20B, RCOR3
18	4	4	6	APBA1, CASK, KCNJ12, LIN7B
19	4	4	6	MRPS2, MRPS31, MRPS5, PTCD3
20	4	4	6	IFT122, IFT43, TULP3, WDR35
21	4	4	6	ARCN1, COPA, COPG1, COPZ1
22	4	4	6	POLE, POLE2, POLE3, POLE4
23	3.882	35	66	ANAPC1, ANAPC10, ANAPC16, ANAPC5, BUB1B, CBX1, CBX3, CDC23, CDC7, CHAF1A, CHAF1B, DBF4, EMSY, EXOC1, EXOC2, EXOC3, EXOC4, EXOC5, EXOC7, GATAD1, KDM5A, KPNA2, KPNB1, MDC1, MYC, NUP153, NUP62, PENK, PHF12, PPP2R5A, PPP2R5D, RBL1, SGO1, SIN3B, SMAD3
24	3.689	46	83	ANAPC4, BUB3, CCDC8, CDC25A, CDC25B, CDC25C, CDC27, CDC37, CUL4A, CUL7, DCAF5, DDA1, FAAP100, FAAP20, FANCB, FANCG, FANCM, IRS1, IRS2, LATS1, LATS2, MAD2L1, MAP3K5, MAPK14, NEK2, NF2, NTRK1, OBSL1, PHLPP2, POLA1, POLA2, PRIM1, PRIM2, RAE1, RASSF8, RB1, SET, STK11, STK4, TTLL1, UBTF, USP12, USP46, WDR20, WDR48, YWHAH
25	3.5	5	7	SNAP23, STX6, STXBP5, VAMP2, VAMP3
26	3.5	5	7	HLA-A, HLA-C, HLA-E, HLA-F, LILRB1
27	3.5	5	7	CCND2, CCND3, CDK4, CDK6, CDKN2C
28	3.357	29	47	ATF7, BBIP1, BBS1, BBS2, BBS7, CDK2, CREB5, DNAJB1, E2F1, FOXO3, HNRNPD, HSPA1A, HSPA4, HUWE1, JUN, JUNB, MAPK9, MCL1, MDM2, MYH9, NFATC2, PPP1CB, PPP1R10, PTBP1, SMAD4, STUB1, TOX4, TRIM33, TTC8
29	3.333	4	5	ASF1A, ASF1B, NASP, TONSL
30	3.333	4	5	DMD, DTNA, SNTA1, SNTB1
31	3.333	4	5	HBA1, HBB, NDUFAF5, PGPEP1
32	3.333	4	5	CDC42EP3, SEPT2, SEPT6, SEPT7
33	3.25	9	13	AMFR, AP2A2, AP2M1, AP2S1, CFTR, DAB2, DERL2, SELENOS, SYVN1
34	3.067	91	138	ACTB, ACTN1, AES, AKAP1, AKAP11, AKT1, APP, AR, ARHGEF7, ATF3, BAX, BCL2L2, BID, BLOC1S1, BLOC1S3, BLOC1S6, CASP8, CDKN1A, CEBPB, CSNK1D, DAG1, DBN1, DBNL, DDIT3, EEA1, ENO2, FGFR1, FN1, GABARAP, GIT1, GIT2, GRB10, HDAC3, HIF1A, HMGA1, HNRNPH1, ITGA5, JAK2, JUP, LGALS8, MAP1LC3A, MAPK8IP1, MAPRE1, MAPT, NCBP1, NR0B2, OPTN, PA2G4, PAK2, PARP1, PARP2, PKM, PMAIP1, PPARG, PRKACA, PRKAR2A, PRKAR2B, PRKDC, PRLR, PRPF31, PTPRK, RAB5A, RABEP1, RABGEF1, RB1CC1, RIPK1, RNF146, S100B, SHB, SIAH2, SIRT2, SNAP29, SNAPIN, SSX2IP, STX3, STX4, STX7, STXBP1, TERF1, TGFB1, TGFB1I1, TGFB2, TGFBR3, TPI1, TUBB3, UBD, VAMP7, VAV1, VCP, XPO1, YWHAE
35	3.043	24	35	ASH2L, CTNND1, CTTN, EGFR, GNAI1, GSTM2, GSTM3, GSTM4, HCFC2, HSP90AB1, IGF1R, KMT2B, KMT2C, MAPK8IP2, MTNR1B, NME1, PHB, RBBP5, RGS4, RRAD, TIAM1, TP53, TUBB, ZBTB33
36	3	3	3	POP4, RBM4B, RPP25
37	3	3	3	CCDC94, PLRG1, PRPF19
38	3	3	3	CNOT3, CNOT7, TNRC6A
39	3	9	12	ACD, ALDH2, ANXA2, FGA, FGB, FGG, LDHA, PLAT, POT1
40	3	3	3	FOXP1, FOXP2, FOXP4
41	3	3	3	VPS26A, VPS29, VPS35
42	3	3	3	CD81, CD9, TSPAN4
43	3	3	3	TMED2, TMED3, TMED4
44	3	3	3	CENPM, CENPO, CENPQ
45	3	3	3	THOC2, THOC3, THOC7
46	3	3	3	EHMT2, H3F3A, KAT2B
47	3	3	3	DDX23, SNRNP40, SNRPD2
48	3	3	3	FAF2, UFD1, VCPIP1
49	3	3	3	CEP135, OFD1, PCM1
50	3	3	3	GOLGA4, NOL11, UTP4
51	3	3	3	CACNA1C, RYR2, SRI
52	3	3	3	EMC1, EMC2, EMC3
53	3	3	3	FHIT, HSPD1, HSPE1
54	3	3	3	SKA1, SKA2, SKA3
55	3	3	3	AKR1C1, AKR1C2, AKR1C3
56	3	3	3	BCKDHA, BCKDHB, PPM1K
57	3	3	3	MAB21L1, MEIS1, PBX1
58	3	3	3	AP3D1, AP3M1, AP3S2
59	3	3	3	MIS18A, MIS18BP1, OIP5
60	3	3	3	C3, CFB, CFP
61	3	3	3	ANKRD55, IFT46, IFT74
62	3	3	3	MTMR1, MTMR2, SBF1
63	3	3	3	LMNA, PCBP1, YWHAZ
64	3	3	3	CUL3, KCTD10, KCTD13
65	3	3	3	MAP3K7, STRADB, XIAP
66	3	3	3	SMC5, SMC6, STN1
67	3	3	3	RAB3IP, TRAPPC10, TRAPPC2
68	3	3	3	SCNN1A, SCNN1G, USP2
69	3	3	3	FLNB, FLNC, OTUD1
70	3	3	3	IFNAR2, IRF9, STAT2
71	3	3	3	CALR, PDIA3, SLC2A1
72	2.868	77	109	ACVR2A, AHCTF1, AP2B1, ATRX, BLNK, BMPR1A, BMPR2, CBX5, CRKL, DDX20, DDX3X, DOK1, EEF1E1, EIF2B1, EIF2B5, EIF4A2, EIF4G3, FKBP4, GDF5, GEMIN6, GPR183, HDAC5, HSP90AA1, IL1R1, IL1RAP, INPP5D, IRAK2, KARS, MRPL16, MRPL37, MRPL38, MRPL47, MRPL48, NEDD8, NIFK, NUP107, NUP205, NUP93, OSBPL10, OSBPL11, OSBPL9, OTUB1, P2RX4, PHB2, PLCG2, PRKD1, PTGER3, PTGES3, QKI, RARS, RBFOX1, RBFOX2, RBMX, RBPMS, RUNX3, SMAD1, SMAD5, SMURF2, SNRPB, SNRPF, SREK1, SRPK1, STAMBP, TAF1A, TAF1B, TAF1C, TBK1, TGFBR1, TOLLIP, UQCRB, UQCRC1, UQCRC2, UQCRQ, VAPA, VAPB, YTHDC1, ZBTB16
73	2.857	8	10	ACTG1, GEMIN5, MLH1, MSH6, SNRPA1, SNRPD1, SNRPD3, SUMO2
74	2.8	6	7	MYO5B, RAB11A, RAB11FIP2, RALBP1, REPS1, REPS2
75	2.667	4	4	DNAAF2, H2AFY2, NUFIP1, SYN1
76	2.56	26	32	ADRB2, ARRDC1, ATP6AP2, ATP6V0D1, ATP6V1D, BCL2, BNIP3, BNIP3L, CA8, CBX7, FAM175B, IGHG1, IGKC, ITPR1, L3MBTL2, MYO1C, NEK6, NEK7, NEK9, PHC3, PSME3, RING1, SNAPC1, SNAPC3, TMEM11, USP20
77	2.491	58	71	ASAP1, ATXN3, CCNL2, CDK13, COG2, COG6, COL6A1, COL9A1, ERCC1, ERCC4, FAM122A, FRS2, FYN, GRB2, IQGAP1, KIT, LMNB1, LYN, MAG, MSH2, MSH3, MYL6, MYL6B, NRBF2, NTRK2, PCNA, PIK3C3, PIK3R4, PINK1, POU2F1, PPP3CA, PRKCB, PRKN, RCAN1, RP2, RRAS, SNX9, SPRY1, SPRY2, SQSTM1, SYNJ1, TMEM185A, TNK2, TNPO2, TOMM20, TOMM22, TOMM40, TUBA1A, TUBB4B, TXNRD1, UBA52, UBE4B, UBL7, USP32, VSIG2, XPA, XRCC5, YWHAB
